# Case Study on an Ethical Dilemma

**DOI:** 10.1192/bjo.2022.351

**Published:** 2022-06-20

**Authors:** Anusha Akella, Kyaw Moe

**Affiliations:** Cheshire and Wirral Partnership NHS Foundation Trust, Wirral /Chester, United Kingdom

## Abstract

**Aims:**

Mr AB is a 58-year-old male with diagnosis of Schizoid Personality disorder. An articulate and intelligent man, AB derived happiness and contentment from his work. Due to workplace conflicts, he was asked to resign several years ago and has not worked since. Mr AB then found a sense of purpose in life by looking after his elderly parents. His parents sadly died a few years ago and since then he has been living on his own. He has never married. AB has one brother who helps him with shopping and groceries. Prior to this admission, AB was admitted once a few years ago when he was diagnosed with Depressive Disorder.

**Methods:**

Mr AB was admitted last year with profound self-neglect. He was detained under Section 2MHA as he wasn't eating and drinking and wasn't engaging with services. With the initial diagnosis being Recurrent Depressive Disorder, AB was commenced on treatment for the same and eventually received ECT, for which he had strongly opposed. Following 6 sessions of ECT, AB bargained with the team that he would start eating and drinking if ECT was stopped and did so as well. He then requested a transfer to a different ward and consultant, with whom he shared that he doesn't agree with our diagnosis of depression or Schizoid personality disorder. AB expressed that he doesn't find his life worth living and wants to be left alone. He strongly believed that his liberty to take decisions about his life is being unfairly taken away by the NHS and accused professionals of trying to protect themselves. No evidence of SMI found at this stage. Following several discussions, AB was discharged home. He however was readmitted within a couple of days’ time by his brother following disengagement, self-neglect and again, no evidence of SMI.

**Results:**

A capacitous patient, in the absence of Serious Mental Illness puts forth an argument that purely because his way of living and his opinions on life and death differ from that of the society, doesn't mean that his rights over his life can be taken away from him. He, however, struggles to acknowledge that as fellow humans we are strongly inclined to intervene and try to stop anyone from taking their own life.

**Conclusion:**

A Challenging case that raises several questions surrounding Medical Ethics. The team is now looking into guardianship to ensure welfare of the patient.

